# Age at Onset and Delays in Diagnosis of Central Disorders of Hypersomnolence Over the Past 30 Years

**DOI:** 10.1111/jsr.70170

**Published:** 2025-08-15

**Authors:** Zhongxing Zhang, Lucie Barateau, Séverine Béziat, Ramin Khatami, Yves Dauvilliers

**Affiliations:** ^1^ Center for Sleep Medicine Sleep Research and Epileptology Barmelweid Switzerland; ^2^ Sleep‐Wake Disorders Unit, Department of Neurology Gui‐de‐Chauliac Hospital, CHU Montpellier France; ^3^ National Reference Centre for Orphan Diseases, Narcolepsy, Idiopathic Hypersomnia, and Kleine‐Levin Syndrome Montpellier France; ^4^ Institute of Neurosciences of Montpellier University of Montpellier, INSERM Montpellier France; ^5^ Department of Neurology Inselspital, Bern University Hospital, University of Bern Bern Switzerland

**Keywords:** age at onset, diagnostic delay, idiopathic hypersomnia, narcolepsy, narcolepsy type 2, orexin/hypocretin

## Abstract

Patients with narcolepsy type 1 (NT1), type 2 (NT2), idiopathic hypersomnia (IH) usually suffer from symptoms for years, even decades, before being diagnosed. We aimed to assess age at onset, age at diagnosis and changes in the diagnostic delays of these patients from 1990 to 2020 in a single centre. Age at onset, age at diagnosis and diagnostic delays of patients with NT1, NT2 and IH were collected at the Reference Narcolepsy Centre, Montpellier–France. Age at onset for each disorder was categorised into three life periods (< 18, 18–25, > 25 years). Diagnostic delays were compared among disorders, taking into account sex, different life periods and time periods. NT1 was diagnosed in 415 patients (242 males), NT2 in 127 patients (68 males) and IH in 289 patients (75 males). Age at onset was not different between disorders (peak between 10 and 20 years in NT1, and 15–20 in IH and NT2). NT1 patients had the shortest diagnostic delays compared to NT2 and IH (median 4, 5 and 8 years respectively). Diagnostic delay is getting shorter in NT1 and IH over the last decades. In patients who started symptoms in childhood, diagnostic delays were the shortest in NT1 and the longest in IH. No sex difference in diagnostic delays was found in NT1 and NT2, but IH females had shorter delays than males. In conclusion, patients with NT1 and IH are diagnosed earlier nowadays compared to the 2000s. Increased public awareness and education efforts should be made to increase knowledge of the diseases and to early identify excessive daytime sleepiness.

## 1 Introduction

Narcolepsy type‐1 (NT1), type‐2 (NT2) and idiopathic hypersomnia (IH) are rare neurological sleep disorders with a common main symptom, excessive daytime sleepiness (EDS) (Khatami et al. 2016; Bassetti et al. 2019). They are classified as central disorders of hypersomnolence (CDH) according to current International Classification of Sleep Disorders (ICSD‐3‐TR) (American Academy of Sleep Medicine 2014). Most patients with CDH have delay periods of up to several years or even decades between symptom onset and diagnosis, leading to a substantial medical and socioeconomic burden and reduction in quality of life (Thorpy and Krieger 2014; Jennum et al. 2012). These diagnostic delays have long been reported in NT1, with several attempts to report factors associated with these delays (Khatami et al. 2016; Jennum et al. 2012; Parkes et al. 1995; Broughton et al. 1997; Dauvilliers et al. 1998; Moldofsky et al. 2000; Furuta et al. 2001; Luca et al. 2013; Morrish et al. 2004). NT1 patients, especially children, may have acute and severe disease manifestations with EDS and other REM sleep‐related symptoms, and can be diagnosed soon (i.e., months or even weeks) after symptom onset. The most well‐known cases with acute NT1 are those triggered by H1N1 influenza virus and/or the Pandemrix vaccination during the 2009–2010 H1N1 pandemic (Bassetti et al. 2019; Dauvilliers et al. 2013; Partinen et al. 2012; Han et al. 2011). It has been hypothesized that the diagnostic delay of narcolepsy would be shortened after this pandemic because of the increased media and public awareness of this rare disease. However, a recent study using the European Narcolepsy Network (EUNN) database reported that the diagnostic delay in NT1 remained stable in the past three decades in Europe (Zhang et al. 2022), despite the increased number of patients during the H1N1 pandemic.

Unlike NT1, no increased incidence of NT2 or IH was reported during the H1N1 pandemic, and whether immunological mechanisms are implicated in these two CDH remains questionable (Barateau et al. 2017). Overall, data on age at disease onset, time to diagnosis in NT2 and IH, and their changes over decades, in both adults and children, are lacking. In this study, we aimed to: (1) characterise and compare the age at onset, age at diagnosis and diagnostic delays in a sample of well‐characterised patients with NT1, NT2 and IH diagnosed in a French National Reference Center for Rare Hypersomnias between 1990 and 2020, and (2) assess the changes in diagnostic delays over time.

## 2 Methods

### 2.1 Patients

We analysed retrospectively the data of 831 patients with information on age of disease onset and a clear diagnosis of a CDH, confirmed at the National Reference Centre for Orphan Diseases, Narcolepsy and Rare Hypersomnias, Montpellier, France, and evaluated in this centre between 1990 and 2020. All patients underwent a comprehensive evaluation, with clinical interviews by sleep experts, a polysomnography (PSG) and multiple sleep latency test (MSLT) at baseline, in drug‐free condition. A subgroup of 446 had a lumbar puncture (LP) to measure cerebrospinal fluid (CSF) orexin‐A/hypocretin‐1 levels by radioimmunoassay (RIA). Additionally, 239 patients with IH also underwent a bedrest protocol (as previously described [Evangelista et al. 2018]) to objectify long sleep time (cut‐off at 19 h/32). The final diagnoses were established by the sleep experts of the sleep centre, according to the ICSD‐3 criteria, including for children cases (American Academy of Sleep Medicine 2014). The diagnostic protocol has remained largely stable over time, with the bedrest protocol introduced in our centre in the late 1990s. Diagnoses made before 2014 were retrospectively reviewed based on updated ICSD‐3 criteria, and lumbar punctures began in the 2000s with the introduction of CSF orexin‐A measurement. None of the patients was sleep‐deprived at the time of evaluation, confirmed by sleep diary and medical interview at least, and actigraphy for doubtful cases. For this cross‐sectional study, only the visit at baseline in the Reference Centre was considered.

### 2.2 Assessment of Age at Onset and Diagnostic Delays

The age at first symptom onset was defined as age at onset of hypersomnolence for NT2 and IH, and age at occurrence of sleepiness and/or cataplexy for NT1. Age at onset for each disorder was further categorised into three life periods: childhood (< 18 y.o.), young adulthood (18–25 y.o.) and later adulthood (> 25 y.o.). The cut‐off age of 25 y.o. to split adulthood into young and later adulthood was established by the Massachusetts Institute of Technology (MIT) Young Adult Development Project (MIT, n.d.), demonstrating that young adulthood is a time of dramatic change in biology (i.e., both mental and physical developments) and environment (e.g., independence, social relationships).

The diagnostic delay was defined as the time interval between the age of diagnosis and the age at first symptom onset. For patients who received the same diagnosis in other sleep centres and in the Montpellier sleep centre, their age at diagnosis was the age at which the first diagnosis was made. For patients who changed diagnosis (e.g., patients diagnosed as NT2 at another sleep centre but finally diagnosed as NT1 at our centre due to low orexin level), the diagnostic age corresponded to the age of diagnosis at Montpellier sleep centre. We stratified our patients into subgroups of different time periods as previously reported in a recent EUNN study (Zhang et al. 2022), that is, 1990–1999, 2000–2009, 2010–2013 and 2014–2020. We also defined patients with short and long delay, according to the distribution of the delay (short delay below the 1st quartile and long delay above the 3rd quartile). The ratio between the numbers of patients with short and long delays in each calendar year was then calculated.

### 2.3 Statistical Analyses

In the absence of normal distribution of diagnostic delay, we opted for non‐parametric tests (Zhang et al. 2022). Data are presented by median and interquartile range (IQR). Figures are violin plots representations, where each point in the violin plots represents one patient. The violin plot combines both box plot and probability density plot. The values of the median of the data are given in the violin plots. The diagnostic delays were compared among the three disorders, among different time periods, and among different life periods within the same disorder using the Kruskal–Wallis test, followed by the post hoc pairwise Dunn test with *p*‐value adjusted by false discovery rate (FDR) method. The diagnostic delay between males and females was compared in each disorder and in each life period using the Wilcoxon rank sum test, respectively. Spearman's correlation analysis was used to assess the correlation between the age of hypersomnolence onset and the diagnostic delay in each disorder. The correlation between the short/long delay ratio and the calendar year was tested using Pearson's correlation. All statistical analyses and modelling were performed using R (version 4.2.3). *p*‐Values < 0.05 indicated significance in all statistical analyses.

## 3 Results

NT1 was diagnosed in 415 patients (242 males, 315 had a LP, all showing low orexin levels, i.e., < 110 pg/mL), NT2 in 127 patients (68 males, 77 had a LP, all showing normal or intermediate orexin levels, i.e., > 110 pg/mL) and IH in 289 patients (75 males, 54 had a LP, all showing normal orexin levels, i.e., > 200 pg/mL). Among IH patients who had a prolonged PSG bedrest recording, 200 (83.7%) were diagnosed with long sleep time and 39 (16.3%) as IH without long sleep time.

### 3.1 Age at Disease Onset and Age at Diagnosis

**FIGURE 1:**
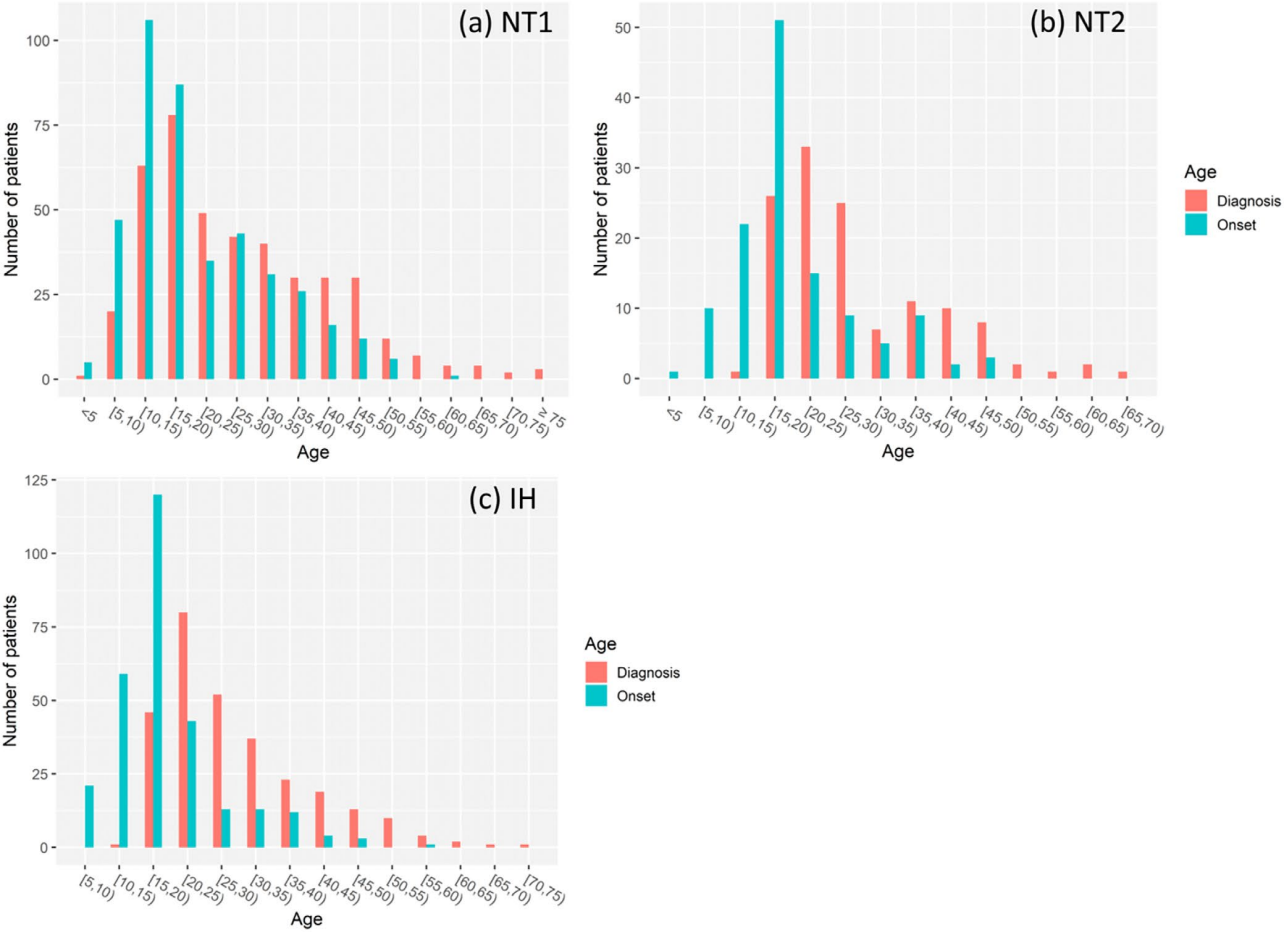
Distributions of the ages of onset and diagnosis in narcolepsy type 1 (NT1) (a), type 2 (NT2) (b) and idiopathic hypersomnia (IH) (c).

The distribution of the ages of first symptom onset and age at diagnosis in patients with NT1, NT2 and IH are shown in Figure 1. In half of NT1, both EDS and cataplexy started at the same age (50.4%); EDS preceded the onset of cataplexy in 40.5%, and cataplexy was the first reported symptom in only 2.7% of patients.

**FIGURE 2:**
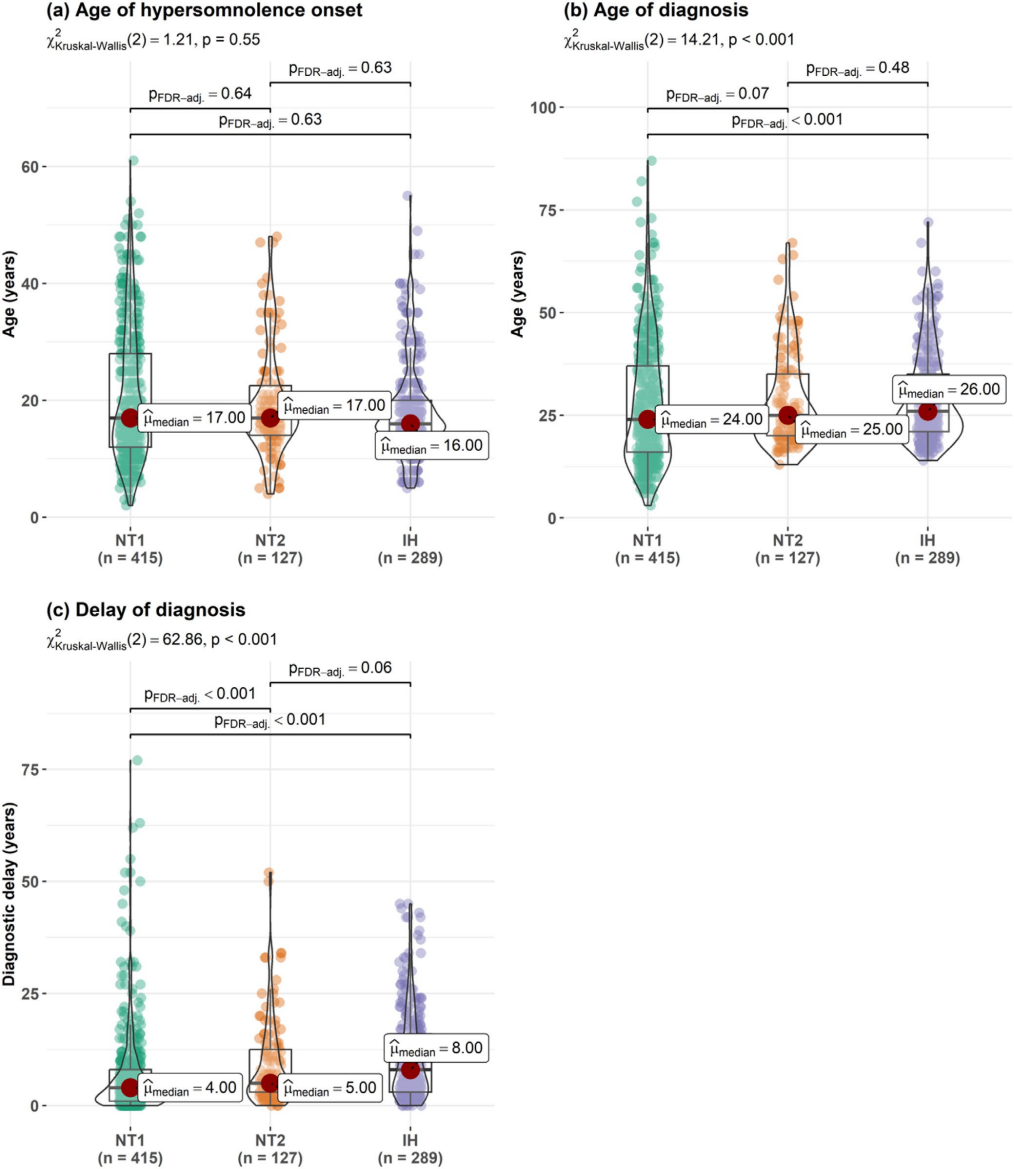
Comparisons of age of onset (a), age of diagnosis (b) and the diagnostic delay (c) among narcolepsy type 1 (NT1), type 2 (NT2) and idiopathic hypersomnia (IH).

**FIGURE 3:**
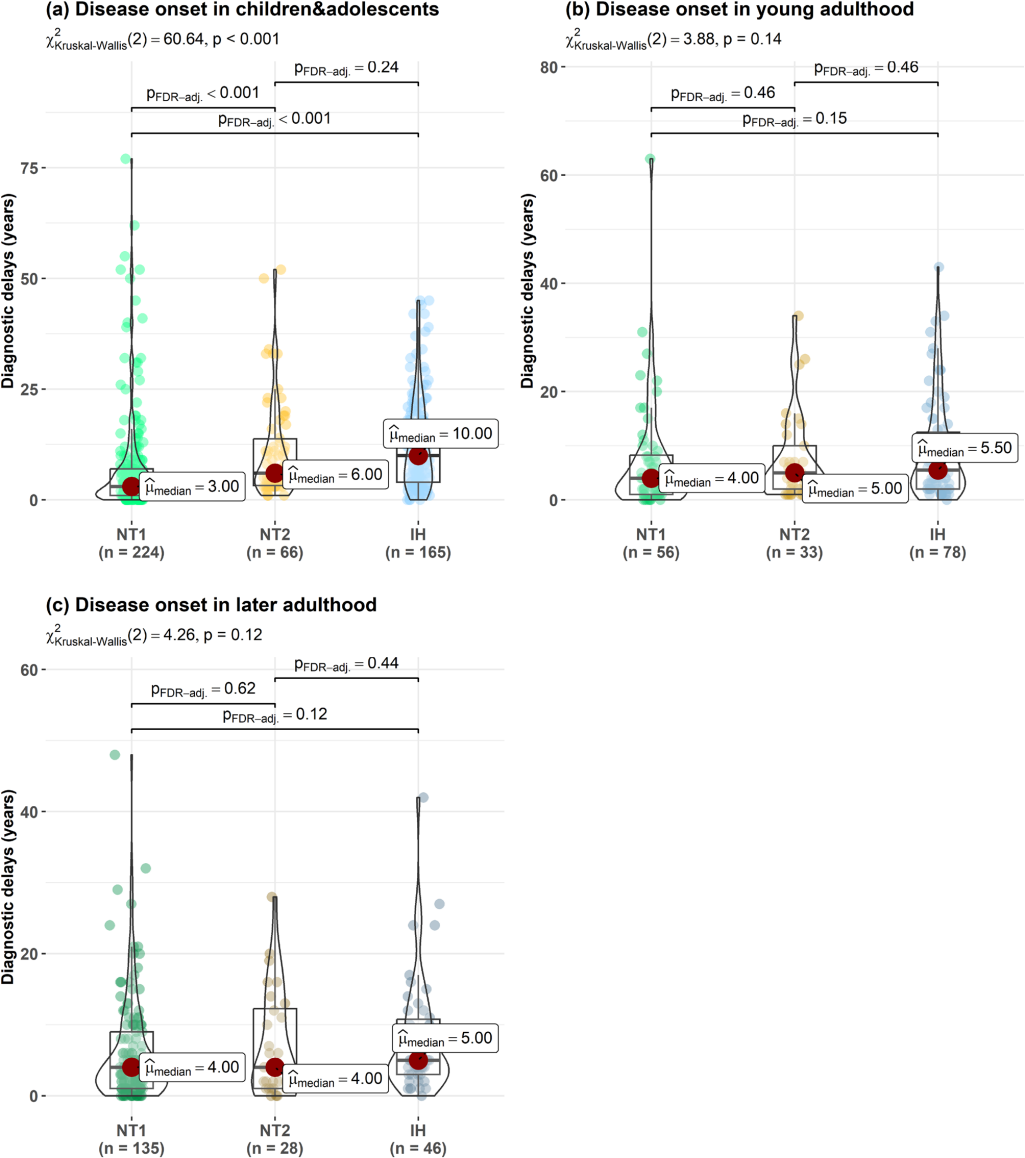
Comparisons of diagnostic delays between different diseases in the three life periods: Subgroup of patients starting symptoms in childhood (a), in young adulthood (b) and in later adulthood (c).

The peak age of disease onset was during the second decade of life, that is, between 10 and 20 y.o. in NT1, and between 15 and 20 y.o. in both IH and NT2. There was no significant difference in the age of symptoms onset among the three disorders (Figure 2). The age of diagnosis did not differ between IH and NT2, but was lower in NT1 compared to IH. Age at onset for each disorder was categorised into three life periods: childhood (< 18 y.o.; 224 NT1, 66 NT2, 165 IH), young adulthood (18–25 y.o.; 56 NT1, 33 NT2, 78 IH) and later adulthood (> 25 y.o.; 135 NT1, 28 NT2, 46 IH). Children with NT1 started the disease at a younger age (median 12 y.o., IQR: 10–15) than children with NT2 (median 14.5 y.o., IQR: 11.3–16, *p* = 0.003) and with IH (median 15 y.o., IQR: 12–16, *p* < 0.001), without a difference between NT2 and IH (Figure 3). There was no difference in the age of onset in adult (i.e., < or > 25 y.o.) patients among the three disorders.

### 3.2 Diagnostic Delays

The mean and median diagnostic delay of all patients with NT1, NT2 and IH was 8.7 and 5 years (IQR: 2–11.5), respectively, with a shorter delay in NT1 (mean: 6.97 years, median: 4 years, IQR: 1–8) compared to NT2 (mean: 9.24 years, median: 5 years, IQR: 3–12.5) and IH (mean: 10.92 years, median: 8 years, IQR: 3–16) (Figure 2). In addition, NT1 patients had a significantly shorter delay than NT2 patients and IH patients when age at onset started below 18 y.o. (Figure 3). There was no difference in the diagnostic delays between the three CDH when symptoms started in young or in later adulthood (Figure 3).

### 3.3 Diagnostic Delays Between Different Life Periods in NT1, NT2 and IH

**FIGURE 4:**
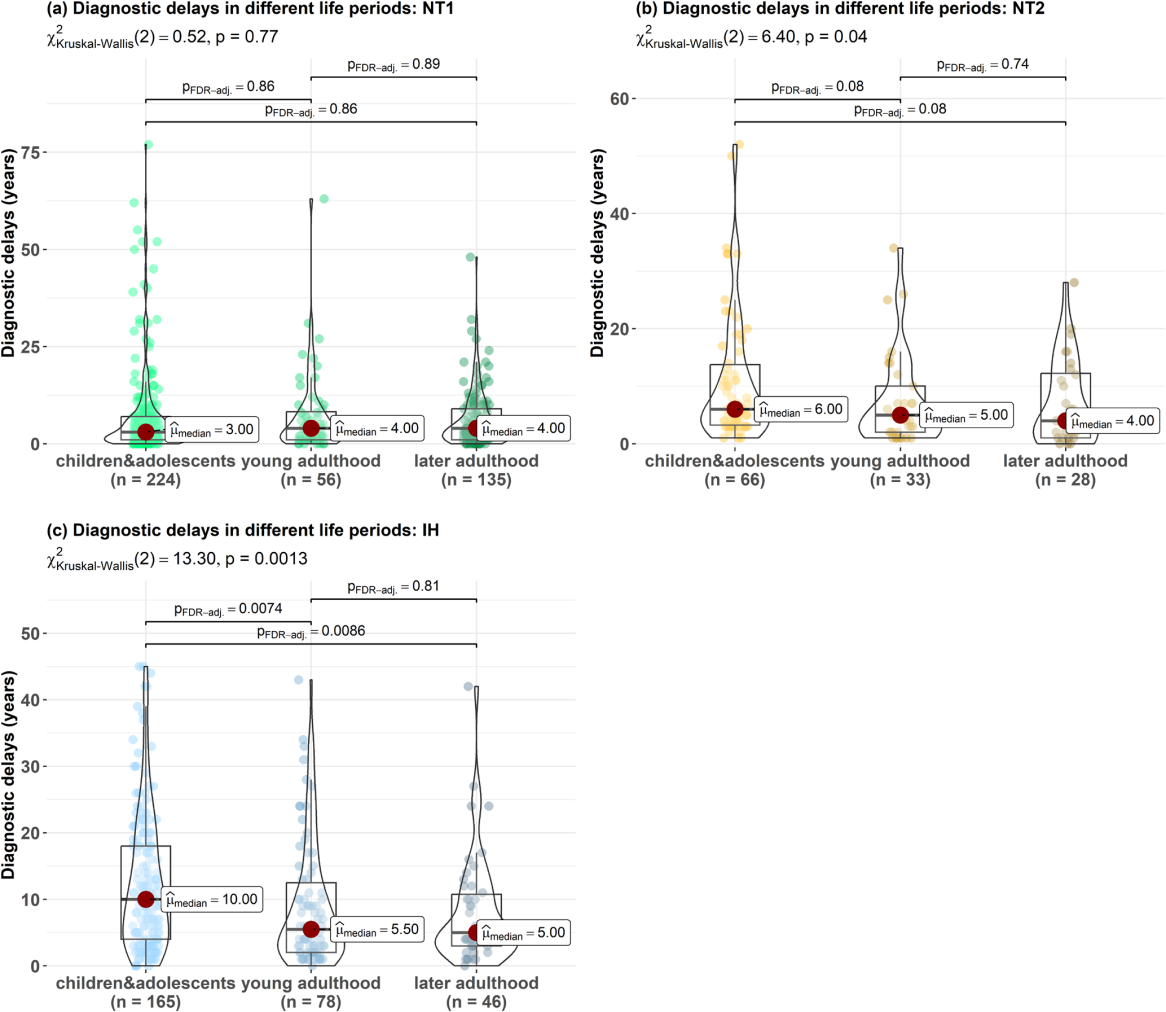
Comparisons of diagnostic delays between different life periods in: Subgroups of patients with narcolepsy type 1 (NT1) (a), type 2 (NT2) (b), and idiopathic hypersomnia (IH) (c).

No differences in the median diagnostic delay were found in NT1 between the three life periods (Figure 4): 3 years when symptoms started in childhood (IQR: 1–7) and 4 years in both young (IQR: 1–8.3) and later adulthood (IQR: 1–9). In NT2, a trend toward shorter delays was found from childhood through young adulthood and to later adulthood (*p* = 0.08): 6 years when symptoms started in childhood (IQR: 3.3–13.8), 5 years in young adulthood (IQR: 2–10) and 4 years in later adulthood (IQR: 1–12.2) (Figure 4). In IH, diagnostic delays were the longest when symptoms started in childhood (10 years, IQR: 4–18), and the shortest in later adulthood (5 years, IQR: 3–10.8), without difference with young adulthood (5.5 years, IQR: 2–12.5) (Figure 4).

We found no sex difference in the diagnostic delays in patients with NT1 (male median: 3 years, IQR: 1–8; female: 4 years, IQR: 1–9; *p* = 0.25) or NT2 (male median: 5 years, IQR: 2.8–14; female: 6 years, IQR: 3–11; *p* = 0.89). By contrast, IH females had shorter delays than males (male median: 11 years, IQR: 4–24; female: 7 years, IQR: 3–13; *p* = 0.0029). Patients with IH with long sleep time had shorter diagnostic delays than IH patients without long sleep time (median 6 years, IQR: 3–13.3 vs. median 11 years, IQR: 3–24, *p* = 0.039).

### 3.4 Changes in Diagnostic Delays From 1990 to 2020

**FIGURE 5:**
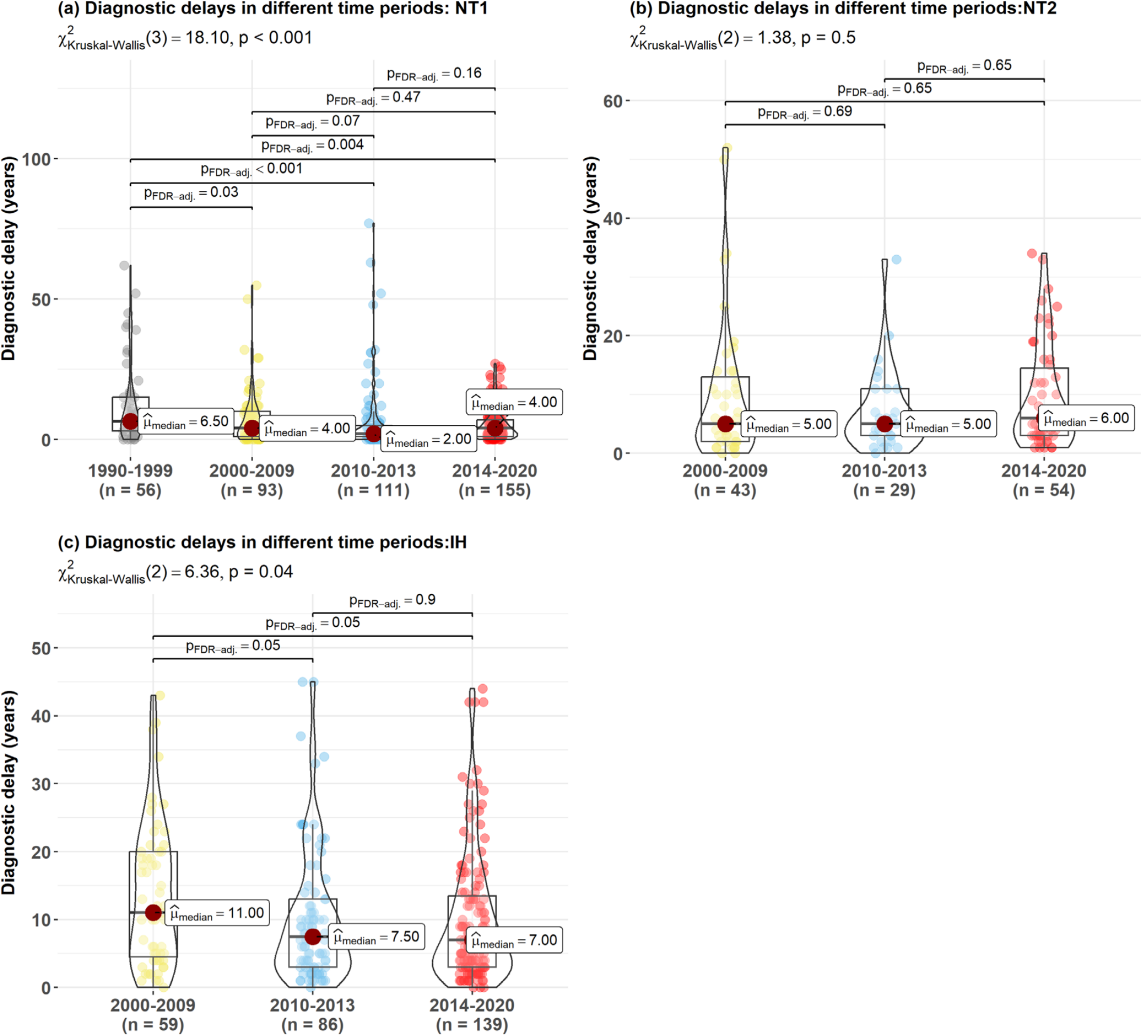
Comparisons of diagnostic delays between different time periods in narcolepsy type 1 (NT1) (a), type 2 (NT2) (b), and idiopathic hypersomnia (IH) (c).

Diagnostic delays in NT1 were getting shorter over the decades (Figure 5), especially during the pandemic period (median delay of only 2 years, IQR: 1–6.6). As only five patients with IH and one patient with NT2 were diagnosed in the 1990s (1990–1999), this period was excluded from the following analyses for both patients with NT2 and IH. Diagnostic delays did not change over time in NT2; while in IH, the diagnostic delays were getting shorter over time (Figure 5).

The diagnostic delay shorter than 2 years and longer than 11.5 years defines short and long delay according to the distribution of the data. The short/long delay ratio correlates to the calendar year in NT1 (*r* = 0.64, *p* = 0.00047), showing a similar trend in IH (*r* = 0.44, *p* = 0.056), but not in NT2 (*r* = 0.069, *p* = 0.82).

The diagnostic delays differed between NT1, NT2 and IH in the 2000s before the 2009–2010 H1N1 pandemic (*p* < 0.001), during (*p* < 0.001) and after the pandemic (*p* < 0.001). Patients with NT1 had significantly shorter diagnostic delays than patients with IH in all time periods (*p* < 0.001 in the three time periods); while they had significantly shorter delays than patients with NT2 only in the 2010s (*p* = 0.03 during 2010–2013, *p* = 0.0003 during 2014–2020). Patients with NT2 had shorter diagnostic delays than patients with IH in the 2000s (*p* = 0.02), but there was no difference in the delays between them in 2010–2013 and 2014–2020.

## 4 Discussion

Our study thoroughly investigated the age at onset, age at diagnosis, and changes in the diagnostic delays in 831 patients with CDH over the last three decades in a National Reference Center for Narcolepsy in France. While age at onset did not differ between the three disorders, we found that NT1 patients with disease onset in childhood had a shorter diagnostic delay compared to those with NT2 or IH. There was no difference in the delay of diagnosis among these disorders when symptoms started later in young or late adulthood. Diagnostic delays have shortened in NT1 over the last two decades, similarly, albeit to a lesser extent, also in IH, whereas the diagnostic delay remained unchanged in NT2. A sex difference is only found in IH patients, that is, females having shorter delays than males.

Contrary to common clinical practice, we did not define the age range of children, young adults, and late adults based on the ages of the patients at the visit to the sleep lab, but defined the different age subgroups according to the age at onset of first symptoms. Current clinical practice would automatically introduce a bias into the delay of diagnosis (Zhang et al. 2022). The definition we proposed allows us to compare the age of patients (children and adults) in different CDHs at the onset of the disease.

The peak age of disease onset has been well studied in NT1 (Dauvilliers et al. 2001) but remained unclear in NT2 and IH. The diagnostic delay in NT1 has also been described in different countries in previous studies (Broughton et al. 1997; Dauvilliers et al. 1998; Luca et al. 2013; Morrish et al. 2004; Zhang et al. 2022; BaHammam and Alenezi 2006; Ingravallo et al. 2012; Frauscher et al. 2013; Ueki et al. 2014; Taddei et al. 2016; Maski et al. 2017; Ohayon et al. 2021; Tio et al. 2018) but rarely in children (at least with such a large sample), and very rarely in NT2 and in IH. Our current study reported that the peak age of disease onset in IH and NT2 was during adolescence (between 15 and 20 y.o.), a particularly vulnerable developmental period for the changes in chronotype (Roenneberg et al. 2004), sleep patterns and insufficient sleep time (Keyes et al. 2015). Remarkably, we found that paediatric patients with NT1 reported a younger age of onset than those with NT2 and IH, which may confirm the different clinical presentation of NT1 with ages (Lividini et al. 2021), and also suggest an earlier manifestation of hypersomnolence in NT1 compared to other CDH diseases.

Our group comparison of diagnostic delays during different life periods revealed differences only in patients starting EDS in childhood and adolescence but not in adulthood. One hypothesis could be differences in the onset and progression of symptomatology between disorders during childhood (Lividini et al. 2021). While NT1 patients can present with an acute/abrupt onset of symptoms (especially cataplexy) and sometimes with a clear triggering factor (Pandemrix vaccine, infection), IH patients often report sleepiness since adolescence or childhood with a gradual onset without clear triggering factors, or they frequently answer to the physician ‘I have always been sleepy’. This insidious course combined with the absence of specific symptom such as cataplexy, the high occurrence of EDS in healthy young adults (due to insufficient sleep and/or changes of chronotype [Roenneberg et al. 2004]), may result in poorer recognition of symptoms by patients themselves and by health care providers. Little is known about NT2 time course and onset, but it has been suggested that an overlap exists between IH and NT2 (Keyes et al. 2015; Fronczek et al. 2020). Poor recognition/misunderstanding of EDS or misdiagnoses of other disorders (e.g., attention deficit hyperactivity disorder [Lecendreux et al. 2015]) might additionally contribute to children/adolescents long delays in NT2 and IH patients. Education about sleepiness and sleep hygiene in childhood/adolescents may be crucial for improving diagnosis of CDH. Shorter diagnostic delays in adults may be due to greater disease impacts on social and work life (e.g., arriving late at work, childcare, driving issues). The finding that IH patients with long sleep time have shorter diagnostic delay than those without long sleep time also favours this explanation.

The diagnostic delays are getting shorter over different time periods, mainly from the 1990s to 2013 in NT1, with the same trend from the 2000s to 2010s in IH; however, no differences were found over time in NT2, potentially due to a lower sample size. These results differ from a previous study using the EUNN database, where the diagnostic delay of NT1 did not change over time (Zhang et al. 2022). This could be explained by the different sources of the data. In the EUNN study, data from multiple centres and different countries were mixed, including those countries where the Pandemrix vaccine was not widely used, whereas in the present study, data were collected from a single French centre that reported a significant increase in NT1 patients during the 2009–2010 H1N1 pandemic (Dauvilliers et al. 2013). The shortest delay in NT1 occurred during the H1N1 pandemic period, with a median delay of 2 years; however, it rebounded to the same level in the 2000s before the pandemic (median delay of 4 years). These results may indicate that the media awareness effect in NT1 is diminishing after the H1N1 pandemic. Therefore, additional efforts are needed to increase public awareness of NT1 and other CDH so that affected patients can be recognised and diagnosed earlier.

The strengths of this study are that the patients are well characterised and all were diagnosed according to the ICSD3 criteria (American Academy of Sleep Medicine 2014) and the predefined standards of a National Reference Center for Narcolepsy and Rare Hypersomnia, specialised in adults and children. A large proportion of patients had a LP to measure CSF orexin levels, adding accuracy to the diagnosis categorisation. Most patients with IH had a long sleep recording in standardised conditions to objectify long sleep time. The ICSD‐3 diagnostic criteria may have contributed to the reduction in diagnostic delays for NT1, as they include the biomarker hypocretin. The bedrest polysomnography protocol developed at our sleep centre may have also contributed to reducing diagnostic delays of IH over time in this study; however, this hypothesis needs to be tested in future studies using data from other sleep centres.

We acknowledge several limitations. First, as a data‐driven study using a clinical database, our results may be influenced by patients' recall bias, especially when diagnostic delays exceed several decades. We minimised this bias by using non‐parametric tests and analysing changes in short/long delay ratios over time (Zhang et al. 2022). Second, our data come from a single centre, and it is unknown whether our results and conclusions can be generalised to other centres or countries. Country differences in the diagnostic delay in NT1 have indeed been reported in previous studies. The sleep unit in Montpellier, as the National Reference Centre, has a specific recruitment process, which may not be representative of other sleep centres in France, particularly those recruiting only paediatric or adult patients. Patients referral pathways from other institutions to Montpellier vary. Some patients self‐refer after online research; others are referred by general practitioners, public or private hospitals, or even by competence or reference centres. Therefore, different socioeconomic backgrounds of patients may influence disease awareness and should be considered in future studies. Our clinical data do not allow for deeper exploration of factors affecting diagnostic delays in CDH or assessment of disease burden and life trajectories among patients with varying delays. We previously reported that the best way to shorten the diagnostic delay is to correctly recognise cataplexy as early as possible (Zhang et al. 2022). Furthermore, the age of onset of cataplexy may have a negative association with the diagnostic delay (Morrish et al. 2004). Indeed, cataplexy may be more difficult to correctly diagnose in children, often misdiagnosed as epilepsy, attention deficit/problems, aggressive behaviour, or attention‐seeking behaviour (Bassetti et al. 2019; Lecendreux et al. 2015; Plazzi et al. 2011; Biscarini et al. 2025). Finally, whether shorter diagnostic delays could impact patient outcomes or early detection of hypersomnolence could lead to improved prognosis and quality of life needs further studies.

In conclusion, this study provides important data on age of disease onset, age of diagnosis, and diagnostic delays in NT2 and IH, two rare CDHs, and carefully compares them relative to NT1 at different life periods and at different time periods over the past decades. Diagnostic delays seem to get shorter over the last decades in NT1 and in IH, but not in NT2. The diagnostic delays remain, however, long in IH and in NT2, especially when symptoms started in childhood. Future studies should identify factors associated with these delays. Increased public awareness and education efforts should be made to increase knowledge of the diseases and to early identify excessive daytime sleepiness, in particular, in children and adolescents.

## Author Contributions

**Zhongxing Zhang:** conceptualization, writing – original draft, methodology, investigation, formal analysis. **Lucie Barateau:** writing – review and editing, data curation, methodology, investigation. **Séverine Béziat:** writing – review and editing, validation, methodology, data curation, investigation. **Ramin Khatami:** conceptualization, methodology, writing – review and editing. **Yves Dauvilliers:** conceptualization, investigation, writing – review and editing, validation, methodology, supervision, data curation.

## Ethics Statement

This study was approved by ethics committees (Comité de Protection des Personnes, France; “*Constitution of a cohort and of a clinical, neurophysiological and biological bank of rare hypersomnolence disorders*” SOMNOBANK).

## Consent

All participants provided written informed consent to participate in the study, with both parents providing consent for minors.

## Conflicts of Interest

We declare no conflicts of interest related to this article. L. Barateau received funds for travelling to conferences by Idorsia, Bioprojet, and board engagements by Jazz, Takeda, Idorsia, and Bioprojet. Y. Dauvilliers received funds for seminars, board engagements, and travel to conferences by UCB Pharma, Jazz, Idorsia, Takeda, Centessa, Avadel, and Bioprojet. S. Béziat reports no disclosure. R. Khatami received funds for travelling to conferences and board engagements by Takeda, Idorsia, and Bioprojet. Z. Zhang has nothing to declare.

## Data Availability

The data that support the findings of this study are available from the corresponding author upon reasonable request.
